# Cardio-metabolic risk in 5-year-old children prenatally exposed to maternal psychosocial stress: the ABCD study

**DOI:** 10.1186/1471-2458-10-251

**Published:** 2010-05-14

**Authors:** Aimée E van Dijk, Manon van Eijsden, Karien Stronks, Reinoud JBJ Gemke, Tanja GM Vrijkotte

**Affiliations:** 1 Department of Public Health, Academic Medical Center - University of Amsterdam, Amsterdam, the Netherlands; 2 Department of Epidemiology, Documentation and Health Promotion, Public Health Service of Amsterdam (GGD), Amsterdam, the Netherlands; 3 Department of Paediatrics, VU University Medical Center, Amsterdam, the Netherlands

## Abstract

**Background:**

Recent evidence, both animal and human, suggests that modifiable factors during fetal and infant development predispose for cardiovascular disease in adult life and that they may become possible future targets for prevention. One of these factors is maternal psychosocial stress, but so far, few prospective studies have been able to investigate the longer-term effects of stress in detail, i.e. effects in childhood. Therefore, our general aim is to study whether prenatal maternal psychosocial stress is associated with an adverse cardio-metabolic risk profile in the child at age five.

**Methods/design:**

Data are available from the Amsterdam Born Children and their Development (ABCD) study, a prospective birth cohort in the Netherlands. Between 2003-2004, 8,266 pregnant women filled out a questionnaire including instruments to determine anxiety (STAI), pregnancy related anxiety (PRAQ), depressive symptoms (CES-D), parenting stress (PDH scale) and work stress (Job Content Questionnaire).

Outcome measures in the offspring (age 5-7) are currently collected. These include lipid profile, blood glucose, insulin sensitivity, body composition (body mass index, waist circumference and bioelectrical impedance analysis), autonomic nervous system activity (parasympathetic and sympathetic measures) and blood pressure.

Potential mediators are maternal serum cortisol, gestational age and birth weight for gestational age (intrauterine growth restriction). Possible gender differences in programming are also studied.

**Discussion:**

Main strengths of the proposed study are the longitudinal measurements during three important periods (pregnancy, infancy and childhood), the extensive measurement of maternal psychosocial stress with validated questionnaires and the thorough measurement of the children's cardio-metabolic profile. The availability of several confounding factors will give us the opportunity to quantify the independent contribution of maternal stress during pregnancy to the cardio-metabolic risk profile of her offspring. Moreover, the mediating role of maternal cortisol, intrauterine growth, gestational age and potential gender differences can be explored extensively. If prenatal psychosocial stress is indeed found to be associated with the offspring's cardio-metabolic risk, these results support the statement that primary prevention of cardiovascular disease may start even before birth by reducing maternal stress during pregnancy.

## Background

Recent evidence, both animal and human, suggests that modifiable, factors during fetal and infant development predispose for cardiovascular disease in adult life and that they may become possible future targets for prevention. The necessity of prevention is growing rapidly with the increasing prevalence of obesity, cardiovascular diseases and metabolic diseases, especially in younger populations [1]. The research proposed in this paper aims at extending the body of evidence on risk factors acting during early life.

Epidemiological and experimental animal studies convincingly show that intrauterine growth restriction is associated with a substantially higher risk of cardiovascular and metabolic disease in adult life, independent from conventional risk factors [2]. Epidemiologist David Barker demonstrated doubled CVD mortality in term born babies that were small for gestational age (SGA) [3]. Other studies also showed that SGA is associated with a substantial part of the risk for adult CVD and its biological forerunners hypertension, impaired glucose tolerance, dyslipidemia and obesity [4-7]. These associations with SGA infants are observed in preterm as well as term born offspring [8].

It is unlikely that low birth weight is the single cause in the chain towards adult CVD. Rather, a common factor is more likely to influence fetal intrauterine growth and simultaneously change the set point of adult physiological systems. This presumed mechanism has been called 'fetal programming', a process whereby a stimulus or insult at a critical point during early development results in permanent adaptation of the organism's structure [9]. Such a potentially involved physiological system is the fetal hypothalamus-pituitary-adrenal (HPA) axis, responsible for stress-regulation and particularly susceptible to programming or reprogramming during fetal or early post natal life [10-13].

The pivotal hormones involved are the hypothalamic corticotrophin releasing hormone (CRH), which stimulates adrenocorticotrophin releasing hormone (ACTH) which in turn modulates the release of cortisol from the adrenals. From here there is a plausible pathway to CVD and the metabolic syndrome via increased exposure of glucocorticoids induced by maternal prenatal psychosocial stress, maternal nutritional stress or exogenous glucocorticoids, to permanent hypertension and glucose intolerance [14-16]. However, so far it is unknown whether maternal prenatal stress exerts sufficient effect to impact the foetus with consequences for CVD risk in later life.

As the HPA-axis is hyperactive due to at least partially preventable sources of exogenous or nutritional maternal stress, it has been suggested that a major focus of CVD prevention should be first trimester pregnancy and the first year of life, when options exist to limit the damage of a mistuned HPA-axis [17]. Such a major shift in focus of primary CVD prevention requires firm human evidence on the full chain of events, which is difficult to obtain [15]. In the last few years, the number of studies extensively studying the effects of exposure to prenatal stress on later life health has been growing [18]. However, few prospective studies relate stress in pregnant women to offspring's birth weight, HPA-axis activity ánd CVD in later life. For example, a study of 396 nulliparous women measured psychosocial stress in the first trimester and subsequently observed stress-related risk of SGA [19]. Wadhwa et al. found that prenatal stress, social support, personality and sociodemographic variables were associated with increased levels of ACTH, beta-endorphin and cortisol [20]. Similar research linked carefully measured sources of stress with SGA, after adjustment of all known competing risks factors for SGA [21]. O'Connor et al. presented human evidence that prenatal anxiety might have lasting effects on HPA axis functioning: They found an association between prenatal anxiety and awakening cortisol in children at age ten [22]. These studies all focused on either pregnancy outcome, thus a more short term measure, or on outcome in the child without including the developmental phases in between.

Additionally, there is evidence indicating a difference in fetal programming effect between boys and girls [23-25]. These studies report that male fetuses are more susceptible to programming; the mechanism responsible is yet to be determined. This gender difference should be looked into more often and in larger study populations.

In the next couple of years, we aim to extend the body of evidence on the association between prenatal maternal stress and cardio-metabolic health in the offspring in later life. In the current paper we describe the design and methods we are planning to use. Therefore, our general aim is:

▪ To study whether prenatal maternal psychosocial stress is associated with an adverse cardio-metabolic risk profile in the child at age five

The corresponding research questions are:

I. Is prenatal maternal stress associated with the child's...

a. lipid profile;

b. blood glucose and insulin sensitivity;

c. body composition;

d. autonomic nervous system activity and blood pressure?

II. Are the associations different for boys and girls?

III. Are the associations mediated by maternal cortisol?

IV. Are the associations mediated by gestational age?

V. Are the associations mediated by birth weight for gestational age (intrauterine growth restriction)?

## Methods/Design

### Study population

**Figure 1:**
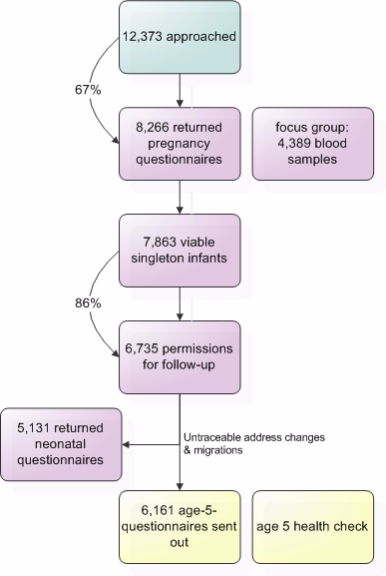
**Procedure of the ABCD Study cohort**.

All measurements are part of a large prospective, multi-ethnic birth cohort, the Amsterdam Born Children and their Development (ABCD) study http://www.abcd-study.nl. This cohort was founded in order to detect and analyze early life factors that are associated with later health and health differences. The progression of the study cohort is presented in a flowchart (figure 1); demographics and characteristics are presented in table 1.

**Table 1:** Study population demographics (mean ± SD) N = 6,161

*Maternal characteristics*		Birth characteristics	
Age (years)	31.0 (± 5.1)	Gender (% girls)	50.1%
Pre-pregnancy BMI (kg/m^2^)	23.0 (± 3.6)	Gestational age (weeks)	39.8 (± 1.7)
Educational level (years after primary school)	9.1 (± 3.9)	% Preterm birth (<37 weeks)	4.9%
% Primipara	55.6%	Birthweight (g)	3452 (± 550)
Ethnicity		% Small for gestational age	11.8%
% Dutch	69.2%		
% Surinamese	5.0%		
% Turkish	3.4%		
% Moroccan	6.1%		
% Other non-western	9.4%		
% Other western	6.9%		
Smoking during pregnancy			
% Not smoking	90.3%		
% 0-5 cigarettes/day	6.3%		
% 6 or more cigarettes/day	3.4%		
% Consuming alcohol	22.9%		

Characteristics of pregnant women and births in the Amsterdam Born Children and their Development (ABCD) Study (the Netherlands)

During phase one (January 2003 - March 2004) all pregnant women from Amsterdam visiting an obstetric care provider were asked to fill out a questionnaire.

Of 12,373 women, 8,266 (67%) did so and a focus group of 4,389 (53%) participants also donated a blood sample for biomarker analysis. A total of 6,735 (81%) women gave permission for follow-up. Three months after birth another questionnaire was sent out and 5,131 (76%) women returned it. In the following years, growth data of the children were collected from Youth Health Care centres.

In 2008, when the children turned five, the current and third phase of the study started. The addresses of 6,161 mothers were retrieved from the Youth Health Care registry; attrition in this follow-up number was largely due to untraceable changes in address or migration. The mothers received a questionnaire, including an informed consent sheet for the age five health check. Data collection is ongoing and children are invited for the health check until December 2010. Two weeks before the health check, mothers receive a notifying letter and an additional self-administered FFQ (Unpublished data: Dutman AE, Stafleu A, Kruizinga A, et al. Validation of a food frequency questionnaire and options for data processing using the doubly labelled water method in children. Submitted, 2009). The health check itself consists of a finger puncture and various health measurements, for which separate consents are signed.

### Independent variable(s): Maternal stress

All the following maternal stress instruments were included in the pregnancy questionnaire.

#### Anxiety

General anxiety was assessed using the Dutch version [26] of the State-Trait Anxiety Inventory (STAI) [27]. The 20 items regarding state-anxiety were included in our questionnaire, with each item scored on a 4-point scale (0 = rarely or none of the time, 1 = some or a little of the time, 2 = occasionally or a moderate amount of the time and 3 = most or all of the time).

#### Pregnancy related anxiety

Pregnancy anxiety was assessed using an abbreviated 10-item version [28] of the Pregnancy Related Anxieties Questionnaire (PRAQ) [29]. Each item is scored on a four-point scale (0 = definitely not true, 1 = not true, 2 = true and 3 = very true). Three aspects that can be distinguished are 'fear of giving birth', 'fear of bearing a physically or mentally handicapped child' and 'concern about one's appearance'.

#### Depressive symptoms

Depressive symptoms were assessed using the validated Dutch version of the 20-item Center for Epidemiological Studies Depression Scale (CES-D) [30,31]. It evaluates the frequency of depressive symptoms experienced over the preceding week. Each item is scored on a four-point scale (0 = rarely or none of the time, 1 = some or a little of the time, 2 = occasionally or a moderate amount of the time and 3 = most or all of the time).

#### Parenting stress

To asses parenting stress a Dutch adaptation [32] of the 20-item Parenting Daily Hassles (PDH) scale was used [33]. The parents rated the occurrence of typical everyday events in parenting and parent-child interactions on a four-point scale (0 = never or rarely, 1 = sometimes, 2 = a lot and 3 = constantly). Our questionnaire did not include the PDH hassle-scale.

#### Work stress

To assess work stress (or job strain) a Dutch version of the Job Content Questionnaire was used [34,35]. It consists of 2 subscales: job demands and job control. The job demands subscale consists of 25 four-point scale items focusing on work pace, mental workload and physical workload. The job control subscale consists of 11 items. Job strain is a combination of high job demands and low control.

### Dependent variables

#### Lipid profile, blood glucose and insulin sensitivity

As a part of the health check, capillary blood is collected in the morning. We use an ambulatory collection kit (Demecal kit: LabAnywhere, Haarlem, The Netherlands) [36] to determine fasting plasma glucose, C-peptide, total cholesterol, high density lipoprotein cholesterol and triglycerides. From these measurements, lipid profile and insulin sensitivity (using the HOMA-IR method [37]) can be derived.

#### Body composition

During the health check measurements, the children are only wearing their underwear bottoms. Height is determined to the nearest millimetre using a Leicester portable height measure (Seca, Hamburg, Germany) and weight to the nearest 100 gram using a Marsden MS-4102 weighing scale (Oxfordshire, United Kingdom). Then waist circumference is measured to the nearest millimetre midway between the costal border and iliac crest, using a Seca measuring tape, and the children lie down for two bioelectrical impedance analysis (BIA) measurements using the Bodystat 1500 MDD system (Bodystat Inc, Douglas, United Kingdom). From these measurements, outcome variables Body Mass Index (kg/m^2^) and fat percentage can be derived [38,39].

#### Autonomic nervous system activity and blood pressure

To measure nervous system effect at the health check we use an ambulatory device, the VU University Ambulatory Monitoring System (VU-AMS; Amsterdam, the Netherlands). Reliability and validity aspects and recording methodology of the VU-AMS have been described previously [40]. The system records three lead electro cardiograms (ECG) and four lead impedance cardiograms (ICG) (Ultratrace Diagnostic ECG with wet gel; ConMed Corporation, Utica, New York, United States of America).

**Figure 2:**
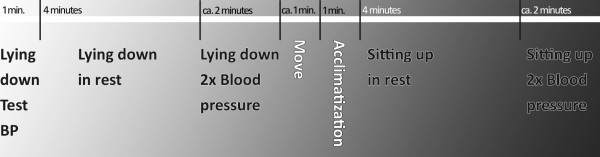
**VU-AMS and blood pressure measurement procedure on a time axis**.

Blood pressure is measured by the Omron 705 IT (Omron Healthcare Inc, Bannockburn, IL, USA) with a small cuff (arm circumference 17-22 cm). The following procedure of VU-AMS and blood pressure measurement is illustrated in figure 2: First, the child lies down in a supine position. During the first minute, one test blood pressure measurement is performed. Then the first official VU-AMS recording period is marked: the child lies in rest for four minutes. The second recording period consists of lying down while blood pressure is measured twice. Then the child is seated at a table and acclimatizes for one minute, followed by four minutes of sitting in rest (recording period 3). Finally, during the fourth recording period, blood pressure is measured twice.

As derivates of parasympathetic nervous system activity, two time domain indices of heart rate variability in the respiratory frequency range, also called respiratory sinus arrhythmia (RSA), will be obtained [41]. The first one is the root-mean-square-of-successive-differences (RMSSD) of the inter beat intervals. This RMSSD is calculated automatically from the R-peaks in the ECG. The second RSA measure is the peak valley estimation (pvRSA) which is obtained automatically by subtracting the shortest inter beat interval during heart rate acceleration in the inspirational phase from the longest inter beat interval during deceleration in the expirational phase.

As a derivate of sympathetic nervous system activity, pre-ejection period (PEP) is used. PEP is the time interval between the onset of ventricular depolarisation (the Q wave onset in the ECG) and the opening of the aortic valves (B-point in ICG) and is considered to be an adequate surrogate measure for sympathic nervous system activity [42]. It will be scored manually in large-scale ensemble averages of the impedance cardiograms [43]. Mean values for each of the four recording periods (figure 2) will be calculated.

### Potential confounders, mediators and effect modifiers

One of the main potential confounders in all analyses is postnatal stress. Depression and anxiety are again assessed in the neonatal questionnaire, using the same tools and scales as in the pregnancy questionnaire. Birth weight, available from Youth Health Care Registration and the Dutch Perinatal Registration (PRN), is considered a potential mediator. It is standardized for gender, gestational age and parity using reference values from the Dutch Perinatal Registration http://www.perinatreg.nl. Gestational age is also studied as a potential mediator.

Maternal characteristics considered potential confounders are age, educational level (years of education after primary school, as a measure of socioeconomic status), ethnicity (country of birth of the mother; definition by the Dutch Central Bureau of Statistics, CBS), smoking (y/n) and alcohol consumption (y/n), all available from the pregnancy questionnaire. Hypertension is also considered a potential confounder (no/pre-existent/pregnancy-induced), available by combining data from the pregnancy questionnaire and Dutch Perinatal Registration (PRN). The categories are classified in accordance with the guidelines of the International Society for the Study of Hypertension in Pregnancy [44].

When the outcome measure is not gender specific, gender of the child is also added as a potential confounder. Gender will also be explored as an effect modifier. Maternal BMI, family history of CVD and physical activity of the child (physical exercise, sedentary behaviour) are available from the age five questionnaire.

When testing the hypothesis regarding body composition, the child's energy intake will be taken into account as a potential mediator or confounder. Total energy intake will be calculated from the food frequency questionnaire.

In all analyses, maternal cortisol is considered a potential mediator. In the focus group of pregnant women (participants of the biomarker study) total cortisol in serum was determined. For each participant, a blood sample was sent to the Regional Laboratory of Amsterdam where 1 ml plasma and serum aliquots were prepared and stored at -80°C until analysis. Total serum cortisol was determined by radio-immunoassay. The interassay coefficient of variation (CV) was 10.2% for low values and 4.9% for high values. In analysis concentrations will be standardized for diurnal variation (sampling took place throughout the day) and gestational age at sampling [45].

### Exclusion criteria

Multiple births are already excluded from the 5-year follow-up of the ABCD cohort. Children using medication known to affect the HPA-axis or autonomic nervous system activity and children with reported heart problems will be excluded from the analyses. This information is available from the age five questionnaire.

### Statistical analyses

For each of the outcome measures correlations with each of the stress scales (continuously when possible) will be explored. For continuous outcome variables ANOVA will be used and for dichotomous or categorical outcome variables Chi^2 ^tests will be used. A total stress score may be calculated by ascribing points to the number of times a mother ends up in the top percentile of a stress scale.

The main analyses are undertaken using linear and/or logistic regression. All associations will be analyzed in a stepwise manner. Step 1: Crude analyses, only adding the independent variable to the model. Step 2: Addition of standardized birth weight and cortisol to explore potential mediation. Step 3: Addition of potential confounders. Step 4: Addition of potential effect-modifiers, by use of interaction terms, which may differ depending on the outcome measure at hand.

For power calculations BMI is chosen as the primary outcome variable. Based on 80% power (1-β), to detect a 0.4 point difference in BMI between children with and without prenatal exposure to high stress (α = 0.05, two-sided), a total of 2,260 children are required. In the calculation we have accounted for the prevalence of high stress, which was based on the job strain data and is estimated at 5% in our population.

### Ethics

Approval of the study was obtained from the Central Committee on Research involving Human Subjects in the Netherlands, the Medical Ethical Committees of participating hospitals, and from the Registration Committee of the Municipality of Amsterdam. Parents provided written informed consent.

## Discussion

The research proposed in this paper aims at extending the body of evidence regarding fetal programming of cardio-metabolic risk in later life, particularly in connection to maternal prenatal stress. In addition to the available non-human studies, the human studies available so far focussed mainly on fragments of the longitudinal chain pregnancy - early life - childhood. Main strengths of the study proposed here are the longitudinal measurements during all three important periods, the extensive measurement of maternal psychosocial stress with validated questionnaires and the thorough measurement of the cardio-metabolic profile in children. The availability of several confounding factors acting during the different periods of early life will give us the opportunity to quantify the independent contribution of maternal stress during pregnancy to the cardio-metabolic risk profile of her offspring. Moreover, the mediating role of intrauterine growth and maternal cortisol can be explored extensively. Also, our large study population allows the exploration of possible gender differences in programming of the cardio-metabolic profile.

Aside from these strengths, some limitations should be acknowledged. Psychosocial stress was assessed only once during pregnancy. On the other hand, the first trimester seems to be particularly important as the HPA-axis of the fetus is most vulnerable for dysregulation during this period [17,46,47]. Plus, this period is considered best for determining stress in pregnant women, because they become decreasingly sensitive to the effects of stress with the advancement of pregnancy [20,48].

Another limitation is the measurement of maternal cortisol which is not optimal because of sampling at different gestational ages and different times of the day. Fortunately we will be able to correct for diurnal rhythm.

If prenatal psychosocial stress is indeed found to be associated with the offspring's cardio-metabolic risk, these results support the statement that primary prevention of CVD may start even before birth by reducing maternal stress during pregnancy.

## Competing interests

The authors declare that they have no competing interests.

## Authors' contributions

AvD drafted the manuscript. TV obtained funding. All authors made substantial contributions to conception, design and revising the manuscript. All authors read and approved the final manuscript.

## Pre-publication history

The pre-publication history for this paper can be accessed here:

http://www.biomedcentral.com/1471-2458/10/251/prepub
